# *MTNR1B* Gene Polymorphisms Are Associated With the Therapeutic Responses to Repaglinide in Chinese Patients With Type 2 Diabetes Mellitus

**DOI:** 10.3389/fphar.2019.01318

**Published:** 2019-11-07

**Authors:** Tao Wang, Xiao-tong Wang, Ran Lai, Hong-wei Ling, Fan Zhang, Qian Lu, Dong-mei Lv, Xiao-xing Yin

**Affiliations:** ^1^Jiangsu Key Laboratory of New Drug Research and Clinical Pharmacy, Xuzhou Medical University, Xuzhou, China; ^2^Department of Pharmacy, the Affiliated Hospital of Xuzhou Medical University, Xuzhou, China; ^3^Department of Endocrinology, the Affiliated Hospital of Xuzhou Medical University, Xuzhou, China

**Keywords:** *MTNR1B*, rs10830963, rs1387153, genetic polymorphism, type 2 diabetes mellitus, repaglinide, pharmacogenetics

## Abstract

The objective of this study was to investigate whether *MTNR1B* gene variants influence repaglinide response in Chinese patients with newly diagnosed type 2 diabetes mellitus (T2DM). A total of 300 patients with T2DM and 200 control subjects were enrolled to identify *MTNR1B* rs10830963 and rs1387153 genotypes by real-time polymerase chain reaction (PCR), with subsequent high-resolution melting (HRM) analysis. Ninety-five patients with newly diagnosed T2DM were randomly selected to undergo 8 weeks of repaglinide treatment (3 mg/day). After 8-week repaglinide monotherapy, patients with at least one G allele of *MTNR1B* rs10830963 showed a smaller decrease in fasting plasma glucose (FPG) (*P* = 0.031) and a smaller increase in homeostasis model assessment for beta cell function (HOMA-B) (*P* = 0.002) levels than those with the CC genotype did. The T allele carriers at rs1387153 exhibited a smaller decrease in FPG (*P* = 0.007) and smaller increases in postprandial serum insulin (PINS) (*P* = 0.016) and HOMA-B (*P* < 0.001) levels compared to individuals with the CC genotype. These data suggest that the *MTNR1B* rs10830963 and rs1387153 polymorphisms are associated with repaglinide monotherapy efficacy in Chinese patients with T2DM.

## Introduction

The incidence of type 2 diabetes mellitus (T2DM) has increased at an alarming rate worldwide over the recent decades (Persaud et al., 2016). It is well established that T2DM is a complex multifactorial and polygenic metabolic disorder characterized by impaired insulin secretion and reduced sensitivity to the peripheral actions of insulin (Pal and McCarthy, 2013; Mannino et al., 2019). Genome-wide association studies (GWAS) have identified more than 150 so-called risk alleles that increase a person’s susceptibility to T2DM, including a common variant in the melatonin receptor 1B gene (*MTNR1B*) (Persaud et al., 2016). The *MTNR1B* gene encodes the melatonin receptor MT2, a G protein-coupled receptor, that is expressed in many tissues, including pancreatic islets (Lyssenko et al., 2008). In 2008, three GWAS in European cohort studies reported that the *MTNR1B* gene was a new susceptibility gene for T2DM (Bouatia-Naji et al., 2008; Lyssenko et al., 2008; Prokopenko et al., 2008). Meanwhile, human and animal studies already pointed to a significant impact of *MTNR1B* on the regulation of glucose homeostasis (Tuomi et al., 2016; Patel et al., 2018; Karamitri and Jockers, 2019). It has been reported that two common variants (rs1387153 and rs10830963) near/in *MTNR1B* were associated with elevated plasma glucose levels, a reduction of the early insulin response to both oral and intravenous glucose, and a faster deterioration of insulin secretion over time (Bouatia-Naji et al., 2008; Lyssenko et al., 2008; Prokopenko et al., 2008; Rönn et al., 2009; Kan et al., 2010). These association studies have demonstrated a link between *MTNR1B* and T2DM, but the fundamental mechanism underlying the association remains far from clear.

Repaglinide is an oral antidiabetic agent that is widely used in the treatment of T2DM. It triggers insulin secretion by closing the ATP-dependent potassium channels and activating Ca^2+^ channels on the surface of pancreatic beta cells, thus lowering plasma glucose levels and especially the postprandial glucose level (Scott, 2012). However, some studies have shown that there were marked interindividual differences in therapeutic response and adverse drug reactions in patients with T2DM treated with repaglinide monotherapy (Eichelbaum et al., 2006; Maruthur et al., 2014; Chen et al., 2016). These differences were partly due to genetic factors related to drug absorption, distribution, metabolism, and targets and genes involved in the causal pathway of T2DM (Tomalik-Scharte et al., 2011; Chakraborty et al., 2015; Chen et al., 2015; Sanchez-Ibarra et al., 2018). Many variants of T2DM susceptibility genes, such as *KCNJ11, ABCC8*, and *KCNQ1*, were associated with pharmacogenomic effects of repaglinide in previous studies (Maruthur et al., 2014). Therefore, polymorphisms in certain susceptibility genes associated with T2DM may potentially affect repaglinide efficacy. However, it remains unknown whether *MTNR1B* single-nucleotide polymorphisms (SNPs) have the same inﬂuence on the therapeutic effects of repaglinide. Thus, in this study, we further explored the association of *MTNR1B* polymorphisms with the therapeutic effect of repaglinide in Chinese patients with T2DM.

## Materials and Methods

### Participants and Study Design

A total of 300 patients with T2DM and 200 healthy controls were recruited for *MTNR1B* polymorphism analysis in this study. All participants were evaluated by analysis of medical histories, physical examinations, and routine clinical laboratory tests. The basic clinical and biochemical characteristics of subjects have previously been described in detail (Wang et al., 2014). T2DM was diagnosed according to the 1999 World Health Organization criteria (Gabir et al., 2000). The inclusion criteria were that the patients had a body mass index (BMI) of 18.5–30 kg/m^2^ and had not received any insulin secretagogue or any agonist or inhibitor of CYP2C8, CYP3A4, and OATP1B1 in the past 3 months. Patients with serious diseases such as acute myocardial infarction, cerebral vascular accident, trauma, and kidney or liver diseases; patients receiving insulin treatment; and pregnant or lactating women were excluded from this study. A total of 95 patients with T2DM (54 male and 41 female) with different *MTNR1B* rs10830963 and rs1387153 genotypes were randomly selected to receive repaglinide (3 mg/day) for 8 consecutive weeks. The study was registered in the Chinese Clinical Trial Registry (registration number: ChiCTR-CCC13003536), and the protocol was approved by the Ethics Committee of the Affiliated Hospital of Xuzhou Medical University. Written informed consent was obtained from each participant before taking part in the study.

### Anthropometric and Biochemical Measurements

The general anthropometric parameters considered for this study were height (in meters), weight (in kilograms), and waist and hip circumferences (in centimeters). After an overnight fast, venous blood samples were obtained both in the fasting state and 2 h later during a standard 75-g oral glucose tolerance test. Parameters were measured at the end of weeks 0 and 8 after administration of repaglinide. Plasma glucose and serum lipids were assessed using standard laboratory methods using a Roche Cobas 8000 analyzer (Roche, Basel, Switzerland). Levels of plasma insulin and hemoglobin A1c (HbA1c) were measured by electro-chemiluminescence assay (Roche, Shanghai, China) and high-performance liquid chromatography assay, respectively. Insulin resistance and beta cell function were calculated with the following formulae: homeostasis model assessment for insulin resistance (HOMA-IR) = fasting insulin level (mU/L) × fasting glucose level (mmol/L)/22.5; homeostasis model assessment for beta cell function (HOMA-B) = 20 × fasting serum insulin (FINS)/(FPG-3.5) (Kong et al., 2017).

### Genotyping

Genomic DNA was extracted from peripheral blood leucocytes. Genotypes for the *MTNR1B* polymorphisms were analyzed using a HRM assay. For the rs10830963 locus, we used the primer pair 5’-GAGGATTTGCTTGCTGAACA-3’ (forward) and 5’-CCCAGGCAGTTACTGGTTCT-3’ (reverse). For rs1387153, we used the primer pair 5’-AACACATGGAAAATGCTTGG-3’ (forward) and 5’-CTGGTTCCAAACCCAACTCT-3’ (reverse). The HRM analyses were performed in a 10 µl reaction system containing 5 µl LC480 HRM Master Mix (Roche Applied Science, Mannheim, Germany), which comprises FastStart Taq DNA Polymerase and the High Resolution Melting Dye in a reaction buffer; 1.2 µl of 25 mM MgCl_2_, 0.2 µl of each primer, 2.5 µl of target DNA sample, 0.5 µl of one known genotype DNA sample (wide type), and 0.4 µl deionized water. The thermal cycling profile for the real-time PCR comprised an initial denaturation at 95°C for 10 min, followed by 55 cycles of 10 s denaturation at 95°C, 10 s annealing at 65°C, and 15 s extension at 72°C. The subsequent melt curve program comprised: 95°C for 1 min, 40°C for 1 min, 70°C for 1 s, 95°C for 1 min, and then cooling at 40°C for 30 s. To conﬁrm the HRM assay results, 5.0% of all samples were directly sequenced.

### Definition of the Response to Repaglinide

Patients were categorized into two cohorts based on glycemic response to repaglinide use: responder and nonresponder. Previous studies have reported that T2DM patients undergoing repaglinide treatment experience a decline of HbA1c by 15%–30% (Abbink et al., 2004; Fang et al., 2014; Jia et al., 2018; Wang et al., 2018). In the current study, levels of HbA1c were reduced by 28% on average. Therefore, responders were defined as those patients with an HbA1c decrease of 25% or more after 8 weeks of repaglinide treatment, whereas nonresponders were defined as patients who failed to achieve this level of decrease.

### Statistical Analysis

Statistical analyses were performed using SPSS software (version 13.0 for Windows; SPSS Inc., Chicago, IL, USA). All data were expressed as mean ± standard deviation (SD), median [interquartile range (IQR)], or percentages as appropriate. The Hardy-Weinberg equilibrium and allelic frequencies in different groups were assessed using χ^2^ tests. A comparison of baseline characteristics in patients with T2DM and healthy controls was carried out using independent samples *t* tests. Differences among three genotypes were analyzed using the one-way ANOVA when appropriate. Paired *t* tests and independent samples *t* tests were used to estimate the effects of repaglinide on biochemical index among genotypes. Parameters with nonnormal distribution were analyzed by the Mann-Whitney *U*-test or the Kruskal-Wallis test. Genotype distribution differences between responders and nonresponders were compared using the χ^2^ test or Fisher’s exact test. Logistic regression analysis was used to test the major determinants of repaglinide response rate. The odds ratio (OR) values were presented with 95% confidence intervals (CIs). Linkage disequilibrium (LD) between SNPs was estimated in control subjects using Haploview version 3.2. Statistical power was calculated by power calculator software (http://www.ncss.com). A value of *P* < 0.05 was considered statistically significant.

## Results

### Genotyping Analysis and Allelic Frequencies

The distribution of genotypes and allele frequencies of the *MTNR1B* rs10830963 and rs1387153 polymorphisms in T2DM patients and control subjects are summarized in **Table 1**. The frequency of the G allele at the *MTNR1B* rs10830963 locus was higher in patients with T2DM than in healthy controls (44.17 vs. 35.75%, *P* = 0.008). For the *MTNR1B* rs1387153 polymorphism, there was no significant difference in allele frequencies between T2DM patients and control subjects. The genotype distributions of rs10830963 and rs1387153 SNPs were in Hardy-Weinberg equilibrium (*P* > 0.05). Assessment of the LD between the polymorphisms using our control subjects revealed a relatively high disequilibrium between rs10830963 and rs1387153 (*r*^2^ = 0.630, *P* < 0.05).

**Table 1:** Genotypes and frequencies of *MTNR1B* polymorphisms in patients with type 2 diabetes mellitus (T2DM) (n = 300) and healthy controls (n = 200).

Genotype	Healthy controls n = 200 (frequency)	T2DM patients n = 300 (frequency)	*P* value
*MTNR1B* rs10830963			
CC	81 (40.50%)	97 (32.33%)	
CG	95 (47.50%)	141 (47.00%)	
GG	24 (12.00%)	62 (20.67%)	0.024
Alleles			
C	257(64.25%)	335 (55.83%)	
G	143 (35.75%)	265 (44.17%)	0.008
*MTNR1B* rs1387153			
CC	79 (39.50%)	109 (36.33%)	
CT	92 (46.00%)	139 (46.33%)	
TT	29 (14.50%)	52 (17.34%)	0.632
Alleles			
C	250 (62.50%)	357 (59.50%)	
T	150 (37.50%)	243 (40.50%)	0.341

### Comparison of Clinical Characteristics Between Different rs10830963 and rs1387153 Genotypes

The baseline clinical characteristics of 300 T2DM patients with different rs10830963 and rs1387153 genotypes are listed in **Tables 2** and **3**. There were no significant differences in sex, age, BMI, waist to hip ratio (WHR), postprandial plasma glucose (PPG), HOMA-IR, triglyceride, total cholesterol, high-density lipoprotein-cholesterol (HDL-C), and low-density lipoprotein-cholesterol (LDL-C) between different genotype groups. However, patients with the GG genotype at *MTNR1B* rs10830963 had higher levels of FPG (*P* < 0.001) but lower levels of FINS (*P* = 0.022), postprandial serum insulin (PINS) (*P* = 0.003), and HOMA-B (*P* < 0.001) than those patients with genotypes CC and CG (**Table 2**, **Figure S1**). The *MTNR1B* rs1387153 polymorphism was markedly associated with levels of FPG and HbA1c; patients with the rs1387153 risk T allele had noticeably higher FPG (*P* = 0.013) and HbA1c (*P* = 0.005) levels compared to patients with the C allele (**Table 3**, **Figure S2**). In addition, the association of *MTNR1B* gene polymorphisms with baseline clinical characteristics was still significant when stratified by age and gender (**Table S1**).

**Table 2:** Baseline characteristics of different genotypes of *MTNR1B* rs10830963 in 300 patients with type 2 diabetes mellitus (T2DM) before treatment with repaglinide.

Parameters	*MTNR1B* rs10830963			Overall *P* value	Adjusted *P* value		
	CC	CG	GG		CC to CG	CG to GG	CC to GG
N (male/female)	97 (58/39)	141 (72/69)	62 (32/30)	0.379			
Age (years)	52.96 ± 7.91	53.46 ± 8.32	51.81 ± 5.69	0.373	1.000	0.481	1.000
BMI (kg/m^2^)	25.99 ± 2.55	25.49 ± 3.07	25.32 ± 2.11	0.239	0.490	1.000	0.393
WHR	0.91 ± 0.05	0.90 ± 0.07	0.91 ± 0.05	0.166	0.061	0.350	0.517
FPG (mmol/L)	9.28 ± 2.48	9.63 ± 2.22	11.11 ± 2.95	<0.001	0.848	<0.001	<0.001
PPG (mmol/L)	16.14 ± 4.65	16.06 ± 4.23	17.92 ± 5.16	0.753	1.000	0.053	0.024
HbA1c (%)	8.99 ± 1.81	9.31 ± 1.58	9.27 ± 1.62	0.303	0.404	1.000	0.877
FINS (mU/L)	9.81 (6.34, 13.84)	7.89 (5.81, 14.28)	7.34 (6.30, 10.01)	0.022	1.000	0.045	0.031
PINS (mU/L)	39.28 (19.86, 53.65)	32.51 (17.56, 51.56)	24.21 (14.61, 33.17)	0.003	0.502	0.004	0.001
HOMA-IR	3.83 (2.57, 5.60)	3.34 (2.40, 5.52)	3.20 (2.63, 4.96)	0.389	1.000	0.547	0.796
HOMA-B	40.13 (21.58, 57.61)	33.27 (17.79, 60.34)	26.31 (16.03, 30.29)	<0.001	0.223	0.005	<0.001
TC (mmol/L)	5.37 ± 1.19	5.15 ± 1.40	5.39 ± 0.95	0.289	0.541	0.636	1.000
TG (mmol/L)	2.25 ± 1.91	2.35 ± 1.92	2.13 ± 1.90	0.745	1.000	1.000	1.000
HDL-C (mmol/L)	1.39 ± 0.39	1.40 ± 0.48	1.47 ± 0.49	0.474	1.000	0.843	0.768
LDL-C (mmol/L)	3.35 ± 1.05	3.27 ± 1.01	3.24 ± 0.86	0.763	1.000	1.000	1.000

BMI, body mass index; WHR, waist to hip ratio; FPG, fasting plasma glucose; PPG, postprandial plasma glucose; HbA1c, glycated hemoglobin; FINS, fasting serum insulin; PINS, postprandial serum insulin; HOMA-IR, homeostasis model assessment for insulin resistance; HOMA-B, homeostasis model assessment for beta cell function; TC, total cholesterol; TG, triglyceride; HDL-C, high-density lipoprotein-cholesterol; LDL-C, low-density lipoprotein-cholesterol. The overall P value reflects the significance before adjustment, and adjusted P value denotes the significance between genotypes following Bonferroni test for multiple comparisons in an additive model (CC vs. CG vs. GG).

**Table 3:** Baseline characteristics of different genotypes of *MTNR1B* rs1387153 in 300 patients with type 2 diabetes mellitus (T2DM) before treatment with repaglinide.

Parameters	*MTNR1B* rs1387153			Overall *P* value	Adjusted *P* value		
	CC	CT	TT		CC to CT	CT to TT	CC to TT
N (male/female)	109 (59/50)	139 (72/67)	52 (31/21)	0.628			
Age (years)	53.21 ± 8.56	52.86 ± 7.54	52.67 ± 6.32	0.901	1.000	1.000	1.000
BMI (kg/m^2^)	25.92 ± 2.60	25.22 ± 2.67	26.04 ± 3.06	0.060	0.129	0.188	1.000
WHR	0.90 ± 0.05	0.90 ± 0.05	0.91 ± 0.10	0.552	1.000	0.836	1.000
FPG (mmol/L)	9.42 ± 2.30	9.82 ± 2.81	10.69 ± 2.14	0.013	0.665	0.105	0.010
PPG (mmol/L)	15.93 ± 4.45	16.82 ± 4.86	16.65 ± 4.25	0.308	0.398	1.000	1.000
HbA1c (%)	8.97 ± 1.48	9.13 ± 1.71	9.87 ± 1.77	0.005	1.000	0.018	0.004
FINS (mU/L)	8.83 (6.34, 13.13)	8.32 (6.30, 12.42)	8.39 (4.81, 14.98)	0.897	1.000	1.000	1.000
PINS (mU/L)	32.51 (17.10, 50.92)	29.43 (19.95, 49.97)	30.22 (12.92, 50.09)	0.894	1.000	1.000	1.000
HOMA-IR	3.46 (2.58, 5.39)	3.37 (2.46, 5.46)	3.35 (2.56, 6.00)	0.770	1.000	1.000	1.000
HOMA-B	34.73 (19.14, 55.56)	30.29 (20.93, 55.14)	22.49 (12.17, 45.31)	0.117	0.896	0.555	0.117
TC (mmol/L)	5.44 ± 1.20	5.18 ± 1.35	5.16 ± 1.09	0.199	0.297	1.000	0.534
TG (mmol/L)	2.11 ± 1.72	2.30 ± 2.01	2.55 ± 2.00	0.384	1.000	1.000	0.517
HDL-C (mmol/L)	1.41 ± 0.39	1.40 ± 0.51	1.42 ± 0.44	0.932	1.000	1.000	1.000
LDL-C (mmol/L)	3.43 ± 1.15	3.16 ± 0.84	3.34 ± 0.99	0.111	0.120	0.823	1.000

BMI, body mass index; WHR, waist to hip ratio; FPG, fasting plasma glucose; PPG, postprandial plasma glucose; HbA1c, glycated hemoglobin; FINS, fasting serum insulin; PINS, postprandial serum insulin; HOMA-IR, homeostasis model assessment for insulin resistance; HOMA-B, homeostasis model assessment for beta cell function; TC, total cholesterol; TG, triglyceride; HDL-C, high-density lipoprotein-cholesterol; LDL-C, low-density lipoprotein-cholesterol. The overall P value reflects the significance before adjustment and adjusted P value denotes the significance between genotypes following Bonferroni test for multiple comparisons in an additive model (CC vs. CT vs. TT).

### Effects of the rs10830963 and rs1387153 Polymorphisms on Therapeutic Efficacy of Repaglinide Treatment in Patients With T2DM

**Figure 1:**
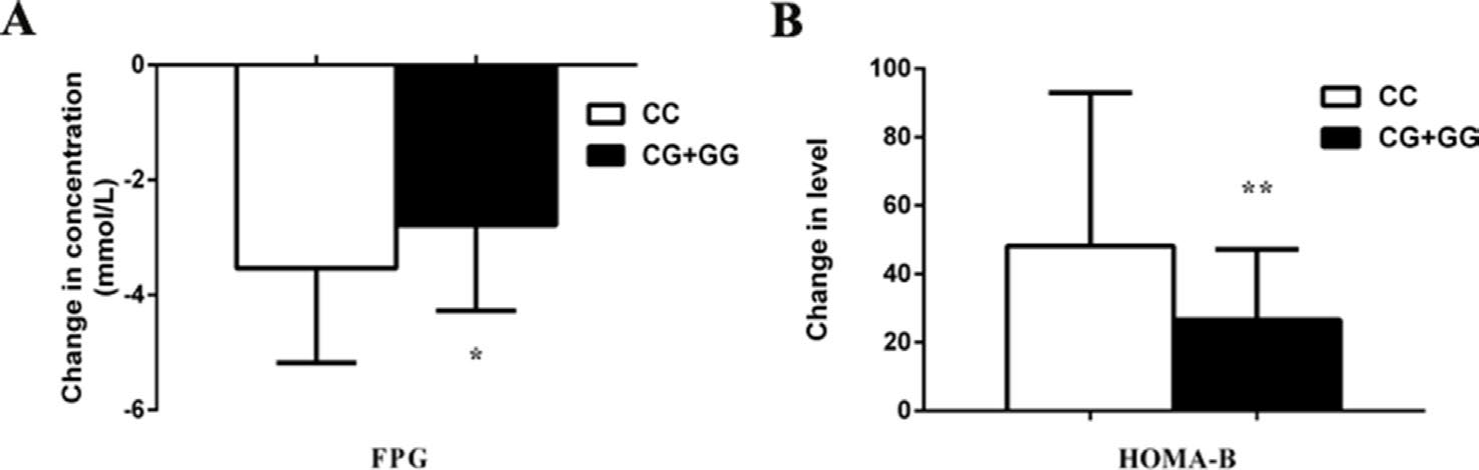
Comparisons of differential values (post-administration levels minus pre-administration levels) of fasting plasma glucose (FPG) **(A)** and homeostasis model assessment for beta cell function (HOMA-B) **(B)** between type 2 diabetes mellitus (T2DM) patients with the CC (n = 44) genotype and those with CG (n = 39) and GG (n = 12) genotypes of *MTNR1B* rs10830963 in T2DM patients after treatment with repaglinide. ^*^P < 0.05 and ^**^P < 0.01 compared with the CC genotype group (n = 95).

**Figure 2:**
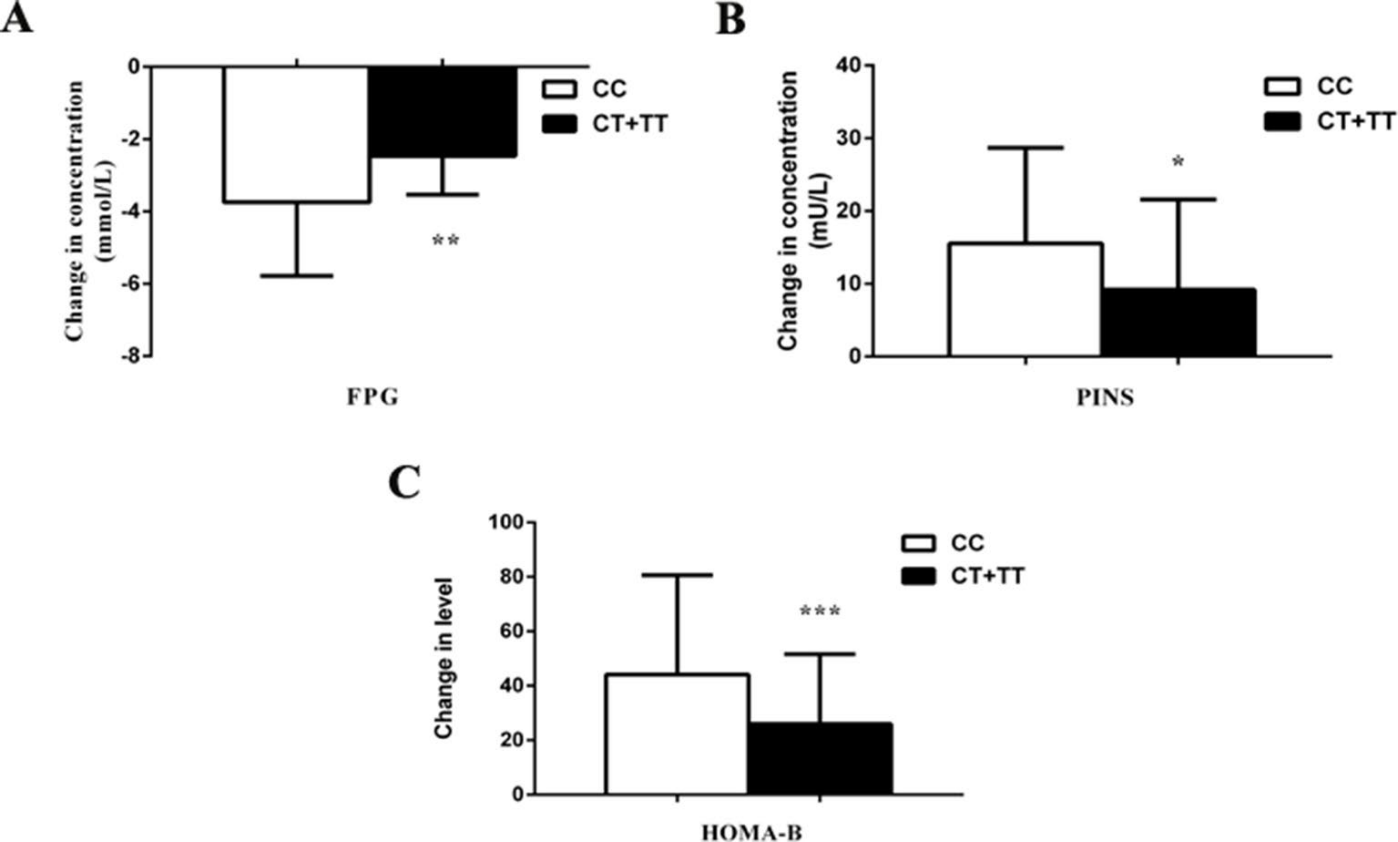
Comparisons of differential values (post-administration levels minus pre-administration levels) of fasting plasma glucose (FPG) **(A)**, postprandial serum insulin (PINS) **(B)**, and homeostasis model assessment for beta cell function (HOMA-B) **(C)** between type 2 diabetes mellitus (T2DM) patients with the CC (n = 47) genotype and those with CT (n = 36) and TT (n = 12) genotypes of *MTNR1B* rs1387153 after treatment with repaglinide. *P < 0.05, **P < 0.01, and ***P < 0.001 compared with the CC genotype group (n = 95).

We observed the effects of *MTNR1B* rs1387153 and rs10830963 on the therapeutic efficacy of repaglinide monotherapy in 95 patients with T2DM. After treatment with 3 mg repaglinide daily for 8 consecutive weeks, the levels of FPG, PPG, HbA1c, HOMA-IR, triglycerides, and total cholesterol decreased significantly, and the levels of FINS, PINS, and HOMA-B markedly increased in all patients with T2DM (**Table S2**). Our data also showed that patients with genotype CC at *MTNR1B* rs10830963 had better efficacy of repaglinide monotherapy with respect to FPG and HOMA-B than CG + GG genotype carriers (**Table S3**, **Figure 1**). Moreover, patients with *MTNR1B* rs1387153 CT + TT genotypes had attenuated efficacy of repaglinide monotherapy with respect to FPG, PINS, and HOMA-B compared with CC genotype carriers (**Table S3**, **Figure 2**). When the patients were stratified by age and gender, the association of *MTNR1B* gene polymorphisms with repaglinide monotherapy efficacy, as measured by FPG, PINS, and HOMA-B, did not vary significantly (**Table S4**).

### Association of *MTNR1B* Genetic Polymorphisms With Response Rate to Repaglinide Treatment

To assess the association of the *MTNR1B* genetic polymorphisms with the response rate to repaglinide treatment in the present study, the genotype and allele frequencies are summarized according to the therapeutic responses in **Table 4**. According to predetermined criteria, the carriers of allele C at the *MTNR1B* rs1387153 locus exhibited good responses to repaglinide therapy; however, there were more responders among the CC allele homozygotes: approximately 70.21% of the CC homozygotes responded compared with only 44.44% of CT heterozygotes and 33.33% of the TT homozygotes (*P* = 0.016). No significant effects of the variation in *MTNR1B* rs10830963 on repaglinide treatment were detected. After adjusting for age, sex, BMI, WHR, FPG, PPG, HbA1c, and blood lipid at baseline, the logistic regression analysis indicated that the *MTNR1B* rs1387153 variant was the only covariate for the response rate to repaglinide treatment. Compared with CC homozygotes, the OR for treatment success was 0.277 for CT (95% CI: 0.088–0.865, *P* = 0.027) and 0.137 for TT (95% CI: 0.027–0.684, *P* = 0.015) genotype carriers.

**Table 4:** Genotype and allele distributions between responders and nonresponders of *MTNR1B* rs1387153 and rs10830963 variants (n = 95).

	Genotype			*P* value	Allele frequency		*P* value
rs1387153	CC	CT	TT		C	T	
Responder (%)	33 (70.21)	16 (44.44)	4 (33.33)		82 (60.08)	24 (40.00)	
Non responder (%)	14 (29.79)	20 (55.56)	8 (66.67)	0.016	48 (36.92)	36 (60.00)	0.003
rs10830963	CC	CG	GG		C	G	
Responder (%)	26 (50.09)	21 (53.85)	6 (50.00)		73 (57.48)	33 (52.38)	
Nonresponder (%)	18 (40.91)	18 (46.15)	6 (50.00)	0.812	54 (42.52)	30 (47.62)	0.505

## Discussion

In the present study, we investigated the potential effects of two SNPs (rs1387153 and rs10830963) near/in *MTNR1B* on the therapeutic efficacy of repaglinide in Chinese patients with T2DM. Our data demonstrated that patients with the G allele of *MTNR1B* rs10830963 or the T allele of rs1387153 may be less responsive to treatment with repaglinide, indicating that the *MTNR1B* genotype may serve as repaglinide response predictor. We therefore suggest that prior genotyping and identification of these individuals may be beneficial for T2DM patients who need treatment with repaglinide.

The common *MTNR1B* gene variant is associated with an increased risk of T2DM across populations of different ethnic backgrounds including Europeans, African-Americans, East Asians, and South Asians (Kim et al., 2011; Ramos et al., 2011). Our study on genetic variants of *MTNR1B* showed that the frequency of the risk G allele of rs10830963 (44.17%) was higher in patients with T2DM than in the control group (*P* < 0.01) and was higher than that in the African (2.70%), European (28.80%), East Asian (42.30%), South Asian (43.00%), and American (19.00%) populations (Auton and Abecasis, 2015). In contrast, rs1387153, which has been identiﬁed as a risk factor in European populations, did not show a signiﬁcant association with T2DM in our subjects, but the frequency of the risk T allele of rs1387153 (40.50%) was lower than that in the East Asian (44.40%) populations and higher than that in the African (38.80%), European (28.70%), South Asian (37.00%), and American (21.00%) populations (Auton and Abecasis, 2015). Our data showed that *MTNR1B* rs10830963 and rs1387153 have dramatically different allele frequencies in Chinese and the other ethnic populations. The major reasons for this discrepancy may be the differences in specific ethnic groups and exposure to environmental factors. The data from this study also showed that patients carrying the rs10830963 risk G allele had higher levels of FPG but lower levels of FINS, PINS, and HOMA-B than those carrying the C allele (**Table 2**, **Figure S1**). In contrast, FPG and HbA1c values were higher in patients with the rs1387153 risk T allele compared to those with the C allele (**Table 3**, **Figure S2**). Robust associations have been identified between genetic variants of the *MTNR1B* gene and increased risk of T2DM along with a higher FPG level and reduced beta-cell function.

The fact that *MTNR1B* is directly or indirectly involved in glucose homeostasis and insulin secretion suggests that *MTNR1B* gene polymorphisms may contribute to interindividual differences in repaglinide response. No previous studies have proven that a patient with a specific *MTNR1B* gene variant shows a better or poorer response to repaglinide. To exclude the effects of *OATP1B1* and *CYP2C8* genetic polymorphisms on the response to repaglinide, 95 patients with different *MTNR1B* genotypes, but the same *OATP1B1* 521TT and CYP2C8*3 139Arg genotypes, were randomly selected from the 300 T2DM patients. In this study, we observed that subjects with at least one G allele of the *MTNR1B* rs10830963 showed a smaller decrease in FPG and a smaller increase in HOMA-B levels than those with the CC genotype, which suggested that the *MTNR1B* rs10830963 G allele confers the poor repaglinide response through a reduction of beta cell function, as measured by HOMA-B. Lyssenko et al. have confirmed the presence of *MTNR1B* in human pancreatic islets and an increased *MTNR1B* mRNA expression in carriers of the rs10830963 risk genotype, reporting a negative correlation between *MTNR1B* mRNA levels and insulin secretion (Lyssenko et al., 2008). Accordingly, overexpression of *MTNR1B* in clonal beta cells results in a stronger inhibition of insulin secretion (Lyssenko et al., 2008). Tuomi and colleagues found that mice with a global deletion of *MTNR1B* exhibit increased insulin release during an intravenous glucose tolerance test when compared to wild-type mice (Tuomi et al., 2016). The proposed mechanism by which *MTNR1B* rs10830963 polymorphism may influence the therapeutic efficacy of repaglinide involves altering the expression of *MTNR1B* in pancreatic beta cells leading to impaired insulin secretion and resulting in deteriorated beta cell function. However, rs10830963 is in the intronic region of *MTNR1B*, and the precise mechanism by which the SNP influences the therapeutic effects of repaglinide is not yet completely clear. Further detailed studies to elucidate the causal variant by fine mapping of the noncoding association signals are warranted.

Regarding rs1387153, which was located 28 kb upstream of the 5’ region of *MTNR1B* at chromosome 11q21-q22, Bouatia et al. conﬁrmed that the rs1387153 T allele was associated with both increased FPG and risk of T2DM in Europeans (Bouatia-Naji et al., 2008). In the present study, the carriers of T alleles at rs1387153 exhibited a smaller decrease in FPG and smaller increases in PINS and HOMA-B levels compared to individuals with the CC genotype following 8 weeks of repaglinide treatment, potentially accounting for the lower failure rate of repaglinide therapy among the CC homozygotes. Further analysis of metabolic traits indicated that the T allele of rs1387153 had negative impacts on FPG and HOMA-B, suggesting that an increased FPG may result, at least in part, from a reduction of beta cell function. The global expression data available from human lymphoblastoid cell lines and human cortical gene expression data have not shown that rs1387153 directly influences *MTNR1B* expression (Dixon et al., 2007; Myers et al., 2007); instead, Lyssenko et al. found that individuals carrying the G allele showed higher expression of *MTNR1B* rs10830963 than carriers of the C allele in human pancreatic islets (Lyssenko et al., 2008). Furthermore, *MTNR1B* rs10830963 was in high LD with rs1387153 (*r*^2^ = 0.630, *P* < 0.05) in our study, as observed in previous studies (Prokopenko et al., 2008; Kan et al., 2010). Therefore, the association of rs1387153 variant with biochemical index and repaglinide efficacy might be involved in the observed LD with rs10830963. Ultimately, detailed functional analyses in pancreatic islets would be necessary to define the causal allele and to confirm that this effect is mediated through altered expression or function of *MTNR1B*.

In interpreting the results of our study, several limitations must be addressed. First, the sample size of included participants was relatively small, although consistent with that used in pilot studies. However, the statistical power of our study (77%–98%) may have been restricted by the sample size. Further studies with a larger sample size and comparisons at multiple time points within 8 weeks are required to conﬁrm the effects of *MTNR1B* polymorphisms on the therapeutic efﬁcacy of repaglinide. Second, the different locations of rs10830963 (in the intronic region of *MTNR1B*) and rs1387153 (in the 5′ region) have made functional studies to elucidate their effects more difficult. The mechanisms by which the two SNPs in *MTNR1B* affect the therapeutic efficacy of repaglinide are not fully understood. Third, incomplete data for healthy controls may have affected a comprehensive exploration of association of *MTNR1B* polymorphisms with clinical characteristics. Fourth, we focused only on the effect of *MTNR1B* gene variants on repaglinide efficacy. However, the possibility still exists that other susceptibility loci for T2DM may affect the therapeutic efficacy of repaglinide. Hence, we will evaluate the joint eﬀects of multiple loci on the efficacy of repaglinide in future studies. Last, although individual differences are the product of the interactions between multiple genetic and environmental factors, it is naturally presumed that some, but not all, patients can benefit from taking certain drugs. We observed distinct responses to repaglinide in different patients with T2DM, which may result from genetic and environmental factors and perhaps other susceptible genetic variations involved in T2DM.

In conclusion, the *MTNR1B* polymorphisms appear to be associated with the therapeutic response to repaglinide in Chinese patients who are newly diagnosed with T2DM. Therefore, the *MTNR1B* risk variants may serve as repaglinide response predictors based on *MTNR1B* mediated beta cell dysfunction. Further pharmacogenetic and functional studies to elucidate the exact effects of *MTNR1B* variants on repaglinide therapeutic efficacy are necessary to lay the foundation for tailoring of a more precise therapy for T2DM.

## Data Availability Statement

All datasets generated for this study are included in the article/**Supplementary Material**.

## Ethics Statement

This study was carried out in accordance with the recommendations of the Ethics Committee of the Affiliated Hospital of Xuzhou Medical University with written informed consent from all subjects. All subjects gave written informed consent in accordance with the Declaration of Helsinki. The protocol was approved by the Ethics Committee of the Affiliated Hospital of Xuzhou Medical University.

## Author Contributions

TW contributed to conception of the article, data acquisition, statistical analysis, result interpretation, manuscript drafting and approved the final version. X-XY contributed to conception of the article, results interpretation, manuscript drafting and approved the final version. X-TW, RL, and H-WL contributed to conception of the article, data acquisition, and approved the final version. FZ contributed to statistical analysis and approved the final version. QL and D-ML contributed to conception of the article, manuscript revising, and approved the final version.

## Funding

This work was supported by the Project Funded by Xuzhou Science and Technology Bureau (KC15SH005).

## Conflict of Interest

The authors declare that the research was conducted in the absence of any commercial or financial relationships that could be construed as a potential conflict of interest.

## Supplementary Material

Figure S1 Baseline levels of FPG, FINS, PINS, and HOMA-B in T2DM patients with CC (n = 97), CG (n = 141) and GG (n = 62) genotypes of *MTNR1B* rs10830963. ^*^, *P*< 0.05 compared with the CG and CC genotype groups (n = 300).

Figure S2 Baseline levels of FPG and HbA1c in T2DM patients with CC (n = 109), CT (n = 139) or TT (n = 52) genotypes of *MTNR1B* rs1387153. ^*^, *P*< 0.05 compared with the CT and CC genotype groups (n = 300).
