# Supplementary figures and images for: MTNR1B Gene Polymorphisms Are Associated With the Therapeutic Responses to Repaglinide in Chinese Patients With Type 2 Diabetes Mellitus
Source: Front Pharmacol. 2019 Nov 7;10:1318. doi: 10.3389/fphar.2019.01318 (PMC6855210; doi:10.3389/fphar.2019.01318)

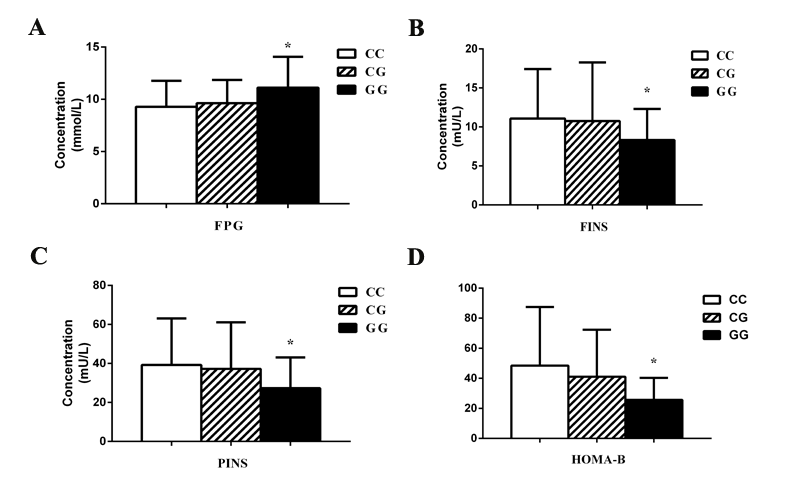

Supplement: Figure S1 — Baseline levels of FPG, FINS, PINS, and HOMA-B in T2DM patients with CC (n = 97), CG (n = 141) and GG (n = 62) genotypes of MTNR1B rs10830963. *, P< 0.05 compared with the CG and CC genotype groups (n = 300). [file Image_1.tif]

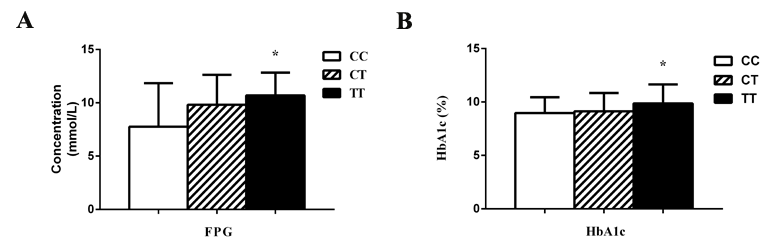

Supplement: Figure S2 — Baseline levels of FPG and HbA1c in T2DM patients with CC (n = 109), CT (n = 139) or TT (n = 52) genotypes of MTNR1B rs1387153. *, P< 0.05 compared with the CT and CC genotype groups (n = 300). [file Image_2.tif]
