# Supplementary material for: MTNR1B Gene Polymorphisms Are Associated With the Therapeutic Responses to Repaglinide in Chinese Patients With Type 2 Diabetes Mellitus
Source: Front Pharmacol. 2019 Nov 7;10:1318. doi: 10.3389/fphar.2019.01318 (PMC6855210; doi:10.3389/fphar.2019.01318)
Supplement: Supplementary file 4 [file Table_1.docx]

**Table S1. Baseline characteristics of different genotypes of *MTNR1B* gene in 300 patients with T2DM stratified by age and gender**

| Parameters | *MTNR1B* rs10830963 | | | | | | | |
| --- | --- | --- | --- | --- | --- | --- | --- | --- |
|  | CC | CG | GG | ^a^*P*  value | CC | CG | GG | ^a^*P* value |
| **Age ≤ 50 years** |  |  |  |  | **Age > 50 years** |  |  |  |
| N (male/female) | 39 (28/11) | 62 (38/24) | 27 (18/9) | 0.552 | 58 (30/28) | 79 (34/45) | 35 (14/21) | 0.466 |
| FPG (mmol/L) | 9.21±2.45 | 10.15±2.34 | 11.50±2.22 | 0.001 | 9.35±2.54 | 9.23±2.04 | 10.79±3.42 | 0.008 |
| PPG (mmol/L) | 15.95±4.92 | 16.42±5.04 | 18.32±4.00 | 0.125 | 16.10±4.45 | 15.77±3.48 | 17.91±5.93 | 0.054 |
| HbA1c (%) | 9.05±1.89 | 9.52±1.70 | 9.43±1.59 | 0.418 | 9.11±1.85 | 9.16±1.46 | 8.86±1.49 | 0.659 |
| FINS (mU/L) | 11.11(7.33,  14.18) | 8.56(5.76,  17.11) | 6.76 (6.09,  9.05) | 0.011 | 9.48(6.31,  13.80) | 7.77(5.85,  13.06) | 7.34 (6.30,  10.11) | 0.161 |
| PINS (mU/L) | 39.17(17.38,  52.10) | 28.64(14.60, 51.42) | 21.80(11.96,33.17) | 0.038 | 39.72 (21.68,  53.77) | 34.51(23.47, 52.93) | 23.25(19.95,30.22) | 0.010 |
| HOMA-IR | 4.47(2.79,  6.27) | 3.30(2.56,  7.08) | 3.37(2.99,  4.94) | 0.769 | 3.82(2.51,  5.45) | 3.34(2.17,  5.44) | 3.14(2.58,  4.94) | 0.296 |
| HOMA-B | 42.81(21.91,  62.71) | 33.25(16.44, 60.10) | 16.21(13.87,27.42) | ＜0.001 | 38.57(23.42,  55.44) | 33.27(17.79, 62.54) | 27.59(22.94,34.73) | 0.022 |
| **Male (n = 162)** | | | | | **Female (n = 138)** | | | |
| FPG (mmol/L) | 9.24±2.26 | 9.46±2.15 | 11.82±3.04 | ＜0.001 | 8.85±2.27 | 9.81±2.29 | 11.01±2.96 | 0.002 |
| PPG (mmol/L) | 16.74±4.41 | 16.03±4.49 | 18.22±3.80 | 0.062 | 15.15±4.72 | 16.08±3.98 | 17.47±4.01 | 0.079 |
| HbA1c (%) | 8.93±1.62 | 9.19±1.51 | 9.51±1.61 | 0.248 | 8.70±2.07 | 9.44±1.65 | 9.49±1.52 | 0.076 |
| FINS (mU/L) | 9.83(6.87,  14.80) | 7.54(5.56,  12.51) | 6.91 (5.42,  10.27) | 0.019 | 10.13(6.30,  13.97) | 8.14(6.00,  17.29) | 7.05 (6.25,  8.32) | 0.015 |
| PINS (mU/L) | 40.29(22.19,  54.06) | 31.29(17.32, 51.18) | 21.80(11.96,33.17) | 0.002 | 39.56(21.70,  53.88) | 32.64(17.56, 54.07) | 23.25(16.14,27.68) | 0.021 |
| HOMA-IR | 4.35 (2.97,  5.98) | 2.99(2.35,  4.80) | 3.37(2.52,  5.01) | 0.075 | 3.82(2.51,  5.22) | 3.78(2.69,  6.40) | 3.00(2.63,  3.95) | 0.241 |
| HOMA-B | 40.40(21.74,  61.29) | 33.27(18.82, 52.70) | 26.96(10.32,27.59) | ＜0.001 | 41.76(25.96,  57.28) | 28.85(15.81, 62.34) | 21.93(13.87,31.40) | ＜0.001 |
| Parameters | *MTNR1B* rs1387153 | | | | | | | |
|  | CC | CT | TT | ^b^*P*  value | CC | CT | TT | ^b^*P* value |
| **Age ≤ 50 years** |  |  |  |  | **Age > 50 years** |  |  |  |
| N (male/female) | 46 (34/12) | 58 (32/26) | 24 (18/6) | 0.076 | 63 (25/38) | 81 (41/40) | 28 (12/16) | 0.408 |
| FPG (mmol/L) | 9.51±2.38 | 10.25±2.68 | 11.12±1.73 | 0.031 | 9.16±2.09 | 9.51±2.88 | 10.77±2.50 | 0.022 |
| PPG (mmol/L) | 16.13±4.99 | 16.63±4.68 | 17.82±4.99 | 0.384 | 15.20±3.54 | 16.96±5.01 | 16.97±4.17 | 0.042 |
| HbA1c (%) | 8.84±1.30 | 9.41±1.92 | 10.21±1.70 | 0.006 | 8.86±1.47 | 8.92±1.52 | 10.03±1.84 | 0.003 |
| FINS (mU/L) | 9.69(6.32,  13.37) | 8.75(6.76,  14.66) | 6.59(4.63,  11.35) | 0.087 | 9.16(7.08,  14.04) | 7.54(5.90,  11.14) | 7.57(4.40,  12.82) | 0.129 |
| PINS (mU/L) | 27.88(16.26, 50.86) | 30.61(18.27, 51.28) | 21.80(9.46, 46.85) | 0.257 | 35.59(23.25, 53.73) | 28.99(21.59, 47.85) | 28.95(11.45,52.42) | 0.223 |
| HOMA-IR | 3.73(2.68,  5.81) | 3.72(2.96,  7.08) | 3.08(2.52,  4.37) | 0.324 | 3.59(2.58,  5.43) | 3.23(1.95,  5.04) | 3.33(2.40,  5.84) | 0.204 |
| HOMA-B | 31.19(17.73, 62.36) | 33.22(14.78, 60.46) | 19.84(11.35,41.69) | 0.017 | 36.33(24.00, 53.90) | 28.85(21.27, 46.67) | 22.28(9.53, 59.57) | 0.207 |
| **Male (n = 162)** | | | | | **Female (n = 138)** | | | |
| FPG (mmol/L) | 9.16±2.00 | 10.09±3.13 | 10.45±1.72 | 0.040 | 9.41±2.38 | 9.60±2.46 | 11.52±2.61 | 0.003 |
| PPG (mmol/L) | 15.68±3.82 | 17.41±5.53 | 17.28±4.91 | 0.110 | 15.26±4.50 | 16.28±3.95 | 17.72±4.25 | 0.308 |
| HbA1c (%) | 8.79±1.23 | 9.12±1.81 | 9.84±1.45 | 0.011 | 8.90±1.66 | 9.20±1.65 | 10.40±1.90 | 0.003 |
| FINS (mU/L) | 9.83(6.42,  14.80) | 8.75(5.81,  10.90) | 7.40(5.08,  16.21) | 0.155 | 8.83(7.08,  13.07) | 8.10(6.40,  15.88) | 6.09(4.40,  11.09) | 0.151 |
| PINS (mU/L) | 37.38(17.24, 51.75) | 31.30(21.52, 44.79) | 27.76(10.79,59.90) | 0.491 | 32.64(21.70, 48.78) | 27.68(19.51, 56.65) | 30.05(14.61,39.30) | 0.372 |
| HOMA-IR | 3.52(2.52,  5.96) | 3.23(2.33,  5.04) | 3.37(2.51,  5.89) | 0.389 | 3.77(2.59,  5.39) | 3.57(2.63,  5.73) | 3.14(2.56,  5.25) | 0.939 |
| HOMA-B | 39.87(22.57, 57.87) | 30.29(20.63, 49.62) | 21.89(12.32,45.92) | 0.084 | 34.73(19.14, 55.76) | 30.29(20.49, 60.81) | 17.90(8.59, 38.19) | 0.238 |

FPG, fasting plasma glucose; PPG, postprandial plasma glucose; HbA1c, glycated hemoglobin; FINS, fasting serum insulin; PINS, postprandial serum insulin; HOMA-IR, homeostasis model assessment for insulin resistance; HOMA-B, homeostasis model assessment for beta cell function.

^a^Additive model: CC vs. CG vs. GG

^b^Additive model: CC vs. CT vs. TT

**Table S2. Clinical characteristics of patients with T2DM** **before and after repaglinide treatment (n=95)**

| Parameters | Before | After | *P* value |
| --- | --- | --- | --- |
| FPG (mmol/L) | 10.17±1.87 | 7.08±1.24 | ＜0.001 |
| PPG (mmol/L) | 16.64±3.54 | 10.89±3.30 | ＜0.001 |
| HbA1c (%) | 9.76±2.04 | 7.02±0.93 | ＜0.001 |
| FINS (mU/L) | 8.20±4.23 | 9.70±4.53 | ＜0.001 |
| PINS (mU/L) | 31.20±16.89 | 43.54±20.42 | ＜0.001 |
| HOMA-IR | 3.63±1.85 | 3.03±1.42 | ＜0.001 |
| HOMA-B | 27.14±15.97 | 62.12±38.66 | ＜0.001 |
| TC (mmol/L) | 5.34±1.41 | 4.90±1.08 | 0.002 |
| TG (mmol/L) | 2.31±1.86 | 1.93±1.78 | 0.030 |
| HDL-C (mmol/L) | 1.44±0.48 | 1.37±0.51 | 0.275 |
| LDL-C (mmol/L) | 3.20±1.08 | 3.14±1.07 | 0.616 |

FPG, fasting plasma glucose; PPG, postprandial plasma glucose; HbA1c, glycated hemoglobin; FINS, fasting serum insulin; PINS, postprandial serum insulin; HOMA-IR, homeostasis model assessment for insulin resistance; HOMA-B, homeostasis model assessment for beta cell function; TC, total cholesterol; TG, triglyceride; HDL-C, high-density lipoprotein-cholesterol; LDL-C, low-density lipoprotein-cholesterol.

**Table S3.** **Comparisons of differential values (DV) in patients with T2DM with different *MTNR1B* genotypes before and after repaglinide treatment**

| Parameters | *MTNR1B* rs10830963 | | ^a^*P* value | *MTNR1B* rs1387153 | | ^b^*P* value |
| --- | --- | --- | --- | --- | --- | --- |
|  | CC | CG+GG |  | CC | CT+TT |  |
| N (male/female) | 44(27/17) | 51(27/24) | 0.409 | 47 (26/21) | 48 (28/20) | 0.767 |
| FPG (mmol/L) |  |  |  |  |  |  |
| Before | 10.25±1.72 | 10.08±1.70 | 0.599 | 10.27±2.14 | 10.07±1.58 | 0.762 |
| After | 6.70±1.20 | 7.48±1.18 | 0.005 | 6.53±0.99 | 7.61±1.22 | ＜0.001 |
| DV | -3.55±1.63 | -2.60±1.24 | 0.031 | -3.74±2.04 | -2.46±1.08 | 0.007 |
| PPG (mmol/L) |  |  |  |  |  |  |
| Before | 16.16±2.57 | 16.85±3.94 | 0.503 | 16.79±3.48 | 16.49±3.62 | 0.646 |
| After | 10.19±2.69 | 11.49±3.67 | 0.083 | 10.59±3.68 | 11.17±2.89 | 0.239 |
| DV | -5.97±3.67 | -5.55±3.82 | 0.588 | -6.19±4.10 | -5.31±3.33 | 0.254 |
| HbA1c (%) |  |  |  |  |  |  |
| Before | 9.79±2.10 | 9.74±2.02 | 0.878 | 9.61±2.16 | 9.91±1.94 | 0.428 |
| After | 6.93±0.80 | 7.10±1.02 | 0.363 | 6.88±0.86 | 7.16±0.98 | 0.148 |
| DV | -2.86±1.86 | -2.63±1.71 | 0.633 | -2.73±1.95 | -2.75±1.61 | 0.754 |
| FINS (mU/L) |  |  |  |  |  |  |
| Before | 8.68±4.25 | 7.61±4.02 | 0.247 | 8.36±3.95 | 8.05±4.52 | 0.462 |
| After | 10.29±4.58 | 9.22±4.38 | 0.204 | 9.89±4.34 | 9.52±4.75 | 0.438 |
| DV | 1.61±4.09 | 1.60±2.48 | 0.617 | 1.53±3.83 | 1.47±3.08 | 0.838 |
| PINS (mU/L) |  |  |  |  |  |  |
| Before | 32.76±18.46 | 28.65±14.45 | 0.395 | 31.25±16.95 | 31.16±17.00 | 0.918 |
| After | 47.46±22.79 | 37.99±16.05 | 0.052 | 46.79±19.77 | 40.36±20.75 | 0.125 |
| DV | 14.70±12.26 | 9.34±8.30 | 0.087 | 15.54±13.13 | 9.20±12.38 | 0.016 |
| HOMA-IR |  |  |  |  |  |  |
| Before | 3.86±1.80 | 3.36±1.80 | 0.165 | 3.72±1.70 | 3.54±2.00 | 0.383 |
| After | 3.00±1.27 | 3.08±1.65 | 0.967 | 2.88±1.36 | 3.17±1.48 | 0.411 |
| DV | -0.86±1.55 | -0.28±1.02 | 0.194 | -0.83±1.46 | -0.38±1.37 | 0.080 |
| HOMA-B |  |  |  |  |  |  |
| Before | 28.25±16.60 | 25.22±14.80 | 0.487 | 27.79±15.94 | 26.50±16.15 | 0.617 |
| After | 76.57±51.07 | 49.77±24.77 | 0.004 | 71.95±38.70 | 52.49±36.51 | 0.001 |
| DV | 48.33±44.73 | 24.55±17.81 | 0.002 | 44.16±36.45 | 25.99±25.65 | ＜0.001 |
| TC (mmol/L) |  |  |  |  |  |  |
| Before | 5.22±1.09 | 5.44±1.65 | 0.555 | 5.19±1.08 | 5.49±1.67 | 0.186 |
| After | 4.91±1.03 | 4.89±1.13 | 0.843 | 4.84±1.03 | 4.96±1.13 | 0.598 |
| DV | -0.31±1.16 | -0.55±1.49 | 0.526 | -0.35±1.11 | -0.53±1.55 | 0.466 |
| TG (mmol/L) |  |  |  |  |  |  |
| Before | 2.48±2.17 | 2.16±1.55 | 0.731 | 2.15±1.46 | 2.47±2.18 | 0.685 |
| After | 1.65±1.28 | 2.09±1.72 | 0.182 | 2.06±2.13 | 1.80±1.37 | 0.028 |
| DV | -0.83±1.37 | -0.07±1.42 | 0.087 | -0.09±1.85 | -0.67±1.46 | 0.512 |
| HDL-C (mmol/L) |  |  |  |  |  |  |
| Before | 1.41±0.45 | 1.48±0.50 | 0.625 | 1.41±0.38 | 1.48±0.55 | 0.649 |
| After | 1.35±0.53 | 1.40±0.49 | 0.583 | 1.35±0.41 | 1.40±0.59 | 0.920 |
| DV | -0.06±0.53 | -0.08±0.69 | 0.820 | -0.06±0.46 | -0.08±0.74 | 0.607 |
| LDL-C (mmol/L) |  |  |  |  |  |  |
| Before | 3.27±0.97 | 3.14±1.17 | 0.387 | 3.28±0.93 | 3.13±1.21 | 0.473 |
| After | 3.22±1.08 | 3.07±1.08 | 0.709 | 3.09±1.06 | 3.19±1.10 | 0.646 |
| DV | -0.05±1.18 | -0.07±1.26 | 0.949 | -0.19±1.20 | -0.06±1.24 | 0.304 |

FPG, fasting plasma glucose; PPG, postprandial plasma glucose; HbA1c, glycated hemoglobin; FINS, fasting serum insulin; PINS, postprandial serum insulin; HOMA-IR, homeostasis model assessment for insulin resistance; HOMA-B, homeostasis model assessment for beta cell function; TC, total cholesterol; TG, triglyceride; HDL-C, high-density lipoprotein-cholesterol; LDL-C, low-density lipoprotein-cholesterol.

^a^Dominant model: CC vs. (CG + GG).

^b^Dominant model: CC vs. (CT + TT).

**Table S4.** **Comparisons of differential values (DV) in patients with T2DM with different *MTNR1B* genotypes before and after repaglinide treatment stratified by age and gender**

| Parameters | *MTNR1B* rs10830963 | | | | | |
| --- | --- | --- | --- | --- | --- | --- |
|  | CC | CG+GG | ^a^*P* value | CC | CG+GG | ^a^*P* value |
| **Age ≤ 50 years** |  |  |  | **Age > 50 years** |  |  |
| FPG (mmol/L) | -3.78±1.71 | -2.70±1.15 | 0.009 | -3.45±1.36 | -2.30±1.29 | 0.009 |
| PPG (mmol/L) | -6.04±3.48 | -5.72±3.56 | 0.735 | -6.20±3.85 | -4.71±4.30 | 0.264 |
| HbA1c (%) | -2.82±1.97 | -2.98±1.65 | 0.749 | -2.92±1.72 | -2.24±1.73 | 0.220 |
| FINS (mU/L) | 1.60±3.82 | 1.75±2.46 | 0.861 | 1.54±4.64 | 1.50±2.50 | 0.973 |
| PINS (mU/L) | 16.07±12.93 | 9.86±7.64 | 0.038 | 15.59±11.60 | 6.58±6.82 | 0.008 |
| HOMA-IR | -0.89±1.66 | -0.28±1.05 | 0.109 | -0.90±1.39 | -0.21±0.96 | 0.085 |
| HOMA-B | 48.37±29.15 | 23.86±19.02 | 0.001 | 51.22±61.98 | 23.22±16.56 | 0.040 |
| **Male (n = 54)** | | |  | **Female (n = 41)** | | |
| FPG (mmol/L) | -3.66±1.71 | -2.79±1.33 | 0.046 | -3.33±1.47 | -2.43±1.15 | 0.036 |
| PPG (mmol/L) | -5.95±3.52 | -5.51±4.53 | 0.692 | -6.03±4.11 | -5.22±3.25 | 0.494 |
| HbA1c (%) | -3.04±2.07 | -2.75±1.89 | 0.598 | -2.48±1.27 | -2.53±1.57 | 0.697 |
| FINS (mU/L) | 1.92±2.87 | 1.79±2.32 | 0.854 | 0.93±6.02 | 1.44±2.65 | 0.817 |
| PINS (mU/L) | 14.56±12.49 | 9.88±8.20 | 0.104 | 15.00±12.20 | 8.86±8.50 | 0.067 |
| HOMA-IR | -0.57±1.10 | -0.28±0.96 | 0.314 | -1.47±2.15 | -0.28±1.09 | 0.159 |
| HOMA-B | 42.72±31.96 | 26.02±19.64 | 0.022 | 60.33±64.11 | 23.24±16.27 | 0.013 |
| Parameters | *MTNR1B* rs1387153 | | | | | |
|  | CC | CT+TT | ^b^*P* value | CC | CT+TT | ^b^*P* value |
| **Age ≤ 50 years** |  |  |  | **Age > 50 years** |  |  |
| FPG (mmol/L) | -3.99±2.02 | -2.60±1.13 | 0.003 | -3.34±2.06 | -2.31±1.02 | 0.042 |
| PPG (mmol/L) | -6.43±3.51 | -5.24±3.43 | 0.213 | -5.80±4.99 | -5.39±3.30 | 0.753 |
| HbA1c (%) | -2.88±2.07 | -2.93±1.48 | 0.922 | -2.48±1.76 | -2.56±1.76 | 0.896 |
| FINS (mU/L) | 1.37±3.75 | 1.98±2.72 | 0.502 | 1.79±4.06 | 0.91±3.39 | 0.456 |
| PINS (mU/L) | 15.12±13.58 | 11.03±11.44 | 0.241 | 16.22±12.72 | 7.21±13.28 | 0.034 |
| HOMA-IR | -0.92±1.61 | -0.32±1.29 | 0.140 | -0.69±1.20 | -0.44±1.47 | 0.556 |
| HOMA-B | 44.99±35.17 | 30.15±31.05 | 0.009 | 42.82±21.46 | 39.42±17.70 | 0.044 |
| **Male (n = 54)** | | |  | **Female (n = 41)** | | |
| FPG (mmol/L) | -3.89±2.06 | -2.47±1.24 | 0.003 | -3.46±1.97 | -2.41±0.83 | 0.007 |
| PPG (mmol/L) | -6.09±4.49 | -5.39±3.36 | 0.523 | -6.17±3.57 | -5.30±3.37 | 0.430 |
| HbA1c (%) | -2.69±2.26 | -3.15±1.64 | 0.398 | -2.63±1.46 | -2.39±1.48 | 0.592 |
| FINS (mU/L) | 1.87±3.41 | 1.48±2.40 | 0.627 | 1.37±4.63 | 1.16±3.46 | 0.870 |
| PINS (mU/L) | 15.95±13.30 | 6.55±11.27 | 0.007 | 14.43±13.03 | 12.60±13.38 | 0.661 |
| HOMA-IR | -0.58±1.24 | -0.37±1.11 | 0.527 | -1.02±1.78 | -0.50±1.62 | 0.334 |
| HOMA-B | 38.95±26.64 | 24.77±24.63 | 0.039 | 51.216±47.74 | 25.64±25.44 | 0.040 |

FPG, fasting plasma glucose; PPG, postprandial plasma glucose; HbA1c, glycated hemoglobin;

FINS, fasting serum insulin; PINS, postprandial serum insulin; HOMA-IR, homeostasis model assessment for insulin resistance; HOMA-B, homeostasis model assessment for beta cell function.

^a^Dominant model: CC vs. (CG + GG).

^b^Dominant model: CC vs. (CT + TT).
